# Birth anthropometry predicts neonatal and infant mortality in rural Bangladesh: a focus on circumferential measurements

**DOI:** 10.1093/ajcn/nqab432

**Published:** 2022-01-11

**Authors:** Yunhee Kang, Lee Shu Fune Wu, Saijuddin Shaikh, Hasmot Ali, Abu Ahmed Shamim, Parul Christian, Alain Labrique, Keith P West

**Affiliations:** Center for Human Nutrition, Department of International Health, Johns Hopkins Bloomberg School of Public Health, Baltimore, MD, USA; Center for Human Nutrition, Department of International Health, Johns Hopkins Bloomberg School of Public Health, Baltimore, MD, USA; The JiVitA Project, Gaibandha, Bangladesh; The JiVitA Project, Gaibandha, Bangladesh; The JiVitA Project, Gaibandha, Bangladesh; James P Grant School of Public Health, BRAC University, Dhaka, Bangladesh; Center for Human Nutrition, Department of International Health, Johns Hopkins Bloomberg School of Public Health, Baltimore, MD, USA; Center for Human Nutrition, Department of International Health, Johns Hopkins Bloomberg School of Public Health, Baltimore, MD, USA; Center for Human Nutrition, Department of International Health, Johns Hopkins Bloomberg School of Public Health, Baltimore, MD, USA

**Keywords:** birth anthropometry, infant mortality, neonatal mortality, newborn circumferential measurements, predictors

## Abstract

**Background:**

Low birth weight predicts risk of infant death. However, several birth measurements may be equally predictive, for which cutoffs and associated risks are less explored.

**Objectives:**

We assessed and optimized population cutoffs of birth length, weight, and midupper arm circumference (MUAC), head circumference (HC), and chest circumference (CC) for predicting neonatal (≤28 d) and infant (≤365 d) mortality in northwest Bangladesh.

**Methods:**

Among 28,026 singletons born in an antenatal micronutrient supplement trial, 21,174 received anthropometry ≤72 h after birth, among whom 583 died in infancy. Optimization for predicting mortality for each measurement was guided by the Youden Index (sensitivity + specificity – 1). Relative risk ratios (RRRs) and positive predictive values (PPVs) were calculated across cutoff ranges for individual and any pair of measurements.

**Results:**

Optimal cutoffs, harmonized to 100-g or 0.5-cm readings, for neonatal and infant mortality were 44.5 cm for length, 2200 g for weight, 9.0 cm for MUAC, 31.0 cm for HC, and 28.5 cm for CC, below which all predicted mortality. However, a CC <28.5 cm, alone and combined with HC <31.0 cm, yielded the highest RRR [9.68 (95% CI: 7.84, 11.94) and 15.74 (95% CI: 12.54, 19.75), respectively] and PPV (11.3% and 10.7%) for neonatal mortality and highest RRR [6.02 (95% CI: 5.15, 7.02) and 9.19 (95% CI: 7.72, 10.95)] and PPV (16.3% and 14.5%) for infant mortality. Pairs of measurements revealed a higher RRR for neonatal and infant mortality than individual measurements of any one pair, although the ranges of PPV remained comparable.

**Conclusions:**

In Bangladesh, multiple birth measurements alone or in combination, particularly chest circumference, predict neonatal and infant mortality.

See corresponding editorial on page 1259.

## Introduction

Newborn anthropometry is employed to assess the adequacy of fetal growth, evaluate nutritional status at birth, and gauge risks of subsequent poor growth, health, development, and survival throughout infancy (1). In rural health care settings of low- to middle-income countries, where births frequently occur at home, weight is most commonly measured, and low birth weight (<2500 g by convention) is the most widely reported anthropometric risk factor for neonatal and postneonatal mortality (2–4). Although less often assessed, a short birth length is also a risk factor (5–7).

Circumferential dimensions, requiring only a tape measure (8), can predict survival. For example, midupper arm circumference (MUAC), often measured in preschool children (9, 10) and occasionally in infancy (11), predicts mortality in both age groups (12, 13) but is rarely assessed at birth. Chest circumference (CC) is uncommonly measured at any age (14, 15), and its ability to predict mortality is unknown. However, when measured at birth, both arm and chest circumferences have been shown to covary with birth weight, with correlation coefficients ranging from 0.63 to 0.96 and 0.55 to 0.91, respectively (14, 16–22), suggesting predictive power.

Head circumference (HC) is a well-established birth dimension (23) that is strongly correlated with length at birth, as well as later in infancy and early childhood (24); the childhood plasma proteome (25); and cognition throughout early school aged years (26–28), but its mortality predictive potential remains unknown. Given simplicity of measurement, low cost, and logistical ease, circumferential measurements at birth may offer as yet unrealized clinical value in primary health care for assessing risks of infant morbidity and mortality.

In Bangladesh, ∼2.9 million live births occur annually (29), and neonatal and infant mortality rates are estimated to be 17 and 25 deaths per 1000 live births, respectively, reflecting 74,000 infant lives lost each year (29). Approximately half of all births in Bangladesh still occur at home (30), usually assisted by primary health care workers or traditional birth attendants who often do not measure birth size due to lack of equipment, training, or realization of its value in predicting survival throughout infancy (31). Estimating and educating primary care workers on the risk of infant mortality associated with individual and combined anthropometric measurements below distinct cutoffs at birth may help motivate their wider adoption to identify and provided extended care to infants at high risk of dying throughout the first year of life.

In this analysis, we explore in a large cohort of Bangladeshi infants the abilities of newborn weight, length, and head, chest, and arm circumferences to predict risks of neonatal (≤28 d) and infant mortality (≤365 d), individually and as any pair of 2 measurements. We identify measurement cutoffs that optimize relative risk ratios (RRRs) and predictive positive values (PPVs), harmonized for both outcomes to facilitate their adoption in primary health care settings of South Asia.

## Methods

### Study population

This study was carried out from 2008 to 2012 in 19 unions in the northwest Bangladesh District of Gaibandha, previously shown to exhibit many characteristics that typify rural Bangladesh (32). The study comprised part of the postnatal assessment protocol for a double-masked, cluster randomized trial (JiVitA-3), conducted from 2008 to 2012, that assessed the efficacy of antenatal multiple micronutrient supplementation compared with iron–folic acid on birth outcomes and infant mortality (33).

### Field procedures

Field procedures of the JiVitA-3 trial have been previously reported (33). Briefly, the study area was divided into 596 comparably populated clusters that served as units of randomization. Married, nonpregnant women of reproductive age were enrolled at the outset while newlyweds were prospectively enrolled throughout the 4-y study. Altogether, 127,282 women were visited at home every 5 wk to detect new pregnancies via a monthly history of amenorrhea confirmed by urine testing. Newly pregnant women were consented; enrolled into the trial; interviewed about socioeconomic status, diet, morbidity, and work patterns; measured for height, weight, and arm circumference; and visited weekly to be given study supplements and monitor pregnancy status.

**FIGURE 1:**
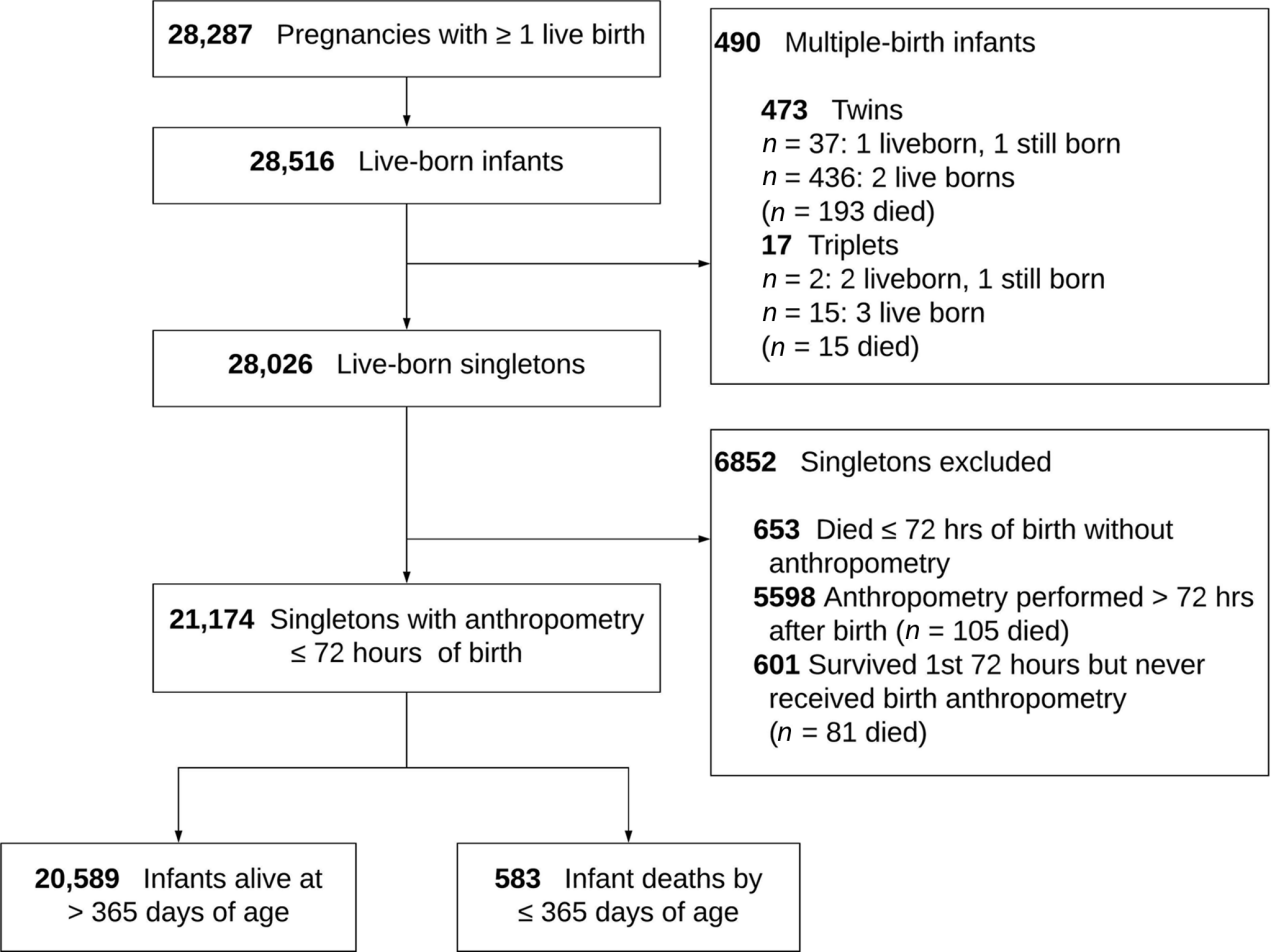
Maternal multiple micronutrient compared with iron–folic acid supplementation trial (JiVitA-3), Gaibandha, Bangladesh, 2008–2012.

Mother–newborn dyads were assessed for vital status, infant anthropometry, and other characteristics shortly after birth and at ∼1, 3, 6, and 12 mo. At birth and subsequent postnatal visits, infant supine length was measured to the nearest 0.1 cm on locally constructed JiVitA length boards; weight was measured to the nearest 10 g (BD585 scales; Tanita Corporation), and HC, CC, and MUAC were measured to the nearest 0.1 cm using Zerfas insertion tapes (8). Of 44,567 pregnant women recruited into the trial, 28,287 delivered ≥1 live-born infants, of whom 28,026 were singletons, among whom 21,174 (75.6%) were measured within 72 h of birth. Among infants who received anthropometry, 328 (1.55%) died within 28 d, and 583 (2.75%) had died by 365 d (**Figure 1**).

Verbal informed consent was obtained from mothers prior to enrollment in the trial. The study protocol was approved by the Johns Hopkins Bloomberg School of Public Health Institutional Review Board, Baltimore, Maryland, and the Bangladesh Medical Research Council, Dhaka, Bangladesh.

### Statistical analysis

The analysis was performed using Stata, version 14.0 (StataCorp LLC). Neonatal, postneonatal, and total infant mortality were defined as deaths occurring within 28 d, 29–365 d, and all deaths through 365 d, respectively, all expressed per 1000 live births. Length-for-age *z* score (LAZ), weight-for-age *z* score, and weight-for-length *z* score (WLZ) of newborns were derived from the WHO international growth standards (34). Baseline maternal and household characteristics and maternal and newborn size were compared for infants who survived the study period (to 365 d) and those who died as neonates (≤28 d) and postneonates (29–365 d). Differences in baseline characteristics and birth outcomes were tested with 1-factor ANOVA for continuous variables and the χ^2^ test for categorical variables, respectively.

Five anthropometric measurements (length, weight, and arm, head, and chest circumferences) were evaluated for their ability to predict the risk of neonatal and infant mortality across their birth measurement range to identify cutoffs optimized for each outcome by logistic regression, accounting for residential neighborhood (“sector,” a study-defined community cluster). Cutoffs were tested incrementally of 10 g for birth weight and 0.1 cm for length and each circumferential measurement. Using the STATA command of “*senspec*,” we estimated sensitivity and specificity at each increment of 10 g for birth weight, 0.1 cm for length, and 0.1 cm for MUAC, HC, and CC across their measurement range. Based on these estimates, we calculated PPVs and negative predictive values. A Youden Index was calculated (sensitivity + specificity – 1), representing the likelihood of measurement being below a specified cutoff in newborns who subsequently died compared with those who survived the neonatal period and first year of life (35). The cutoff for each measurement yielding the highest Youden Index was defined as optimal. Receiver operating characteristic (ROC) curves (36) for sensitivity compared with 1 – specificity were produced for each measurement.

We harmonized the optimal cutoffs by investigator consensus for both neonatal and infant mortality for each measurement as an approach to facilitating application of findings from this study in health care and community assessment settings. Sensitivity and specificity for the combinations of 2 measurements were calculated by 2 × 2 contingency tables (37). Neonatal and infant mortality rates and RRRs based on generalized linear models (38) were calculated for harmonized anthropometric cutoffs for each measurement and combinations of 2 measurements.

A Nelson–Aalen cumulative hazard function was used to draw cumulative hazard curves for neonatal and infant mortality according to each measurement's harmonized cutoff (39).

## Results

This analysis examines the vital experience of 21,174 singletons measured at a median (IQR) age of 16 (8–42) h, among whom 583 died in the first year (Figure 1). A total of 6852 infants were excluded due to having died before the home visit or otherwise not having been assessed within 72 h, groups whose maternal and household characteristics are summarized in **Supplemental Table 1**. Within the analytic cohort, mothers of deceased compared with surviving infants were slightly less educated, literate, and wealthy, as assessed by a locally constructed Living Standards Index (40) (**Table 1**).

**TABLE 1:** Maternal and household characteristics of singletons assessed by anthropometry ≤72 h after birth, by vital status during infancy^1^

Characteristic	Total (*n* = 21,174)		Died				Alive (*n* = 20,591)		*P* value^4^
			Neonatal^2^ (*n* = 328)		Postneonatal^3^ (*n* = 255)				
	*n*	*n* (%) or mean ± SD	*n*	*n* (%) or mean ± SD	*n*	*n* (%) or mean ± SD	*n*	*n* (%) or mean ± SD	
Age, y	21,163	23.1 ± 5.7	328	22.6 ± 5.7	255	23.0 ± 6.0	20,580	23.1 ± 5.6	0.13
Education, y	21,146	4.7 ± 3.4	327	3.5 ± 3.3	253	4.0 ± 3.4	20,566	4.7 ± 4.0	<0.001
Literacy	21,150	12,616 (59.7)	327	177 (54.1)	253	122 (48.2)	20,570	12,317 (59.9)	< 0.001
Reproductive history									
Parity	21,153		327		253		20,573		
0		6994 (33.1)		159 (48.6)		90 (35.6)		6745 (32.8)	< 0.001
1–3		13,047 (61.7)		145 (44.3)		135 (53.4)		12,767 (62.1)	
≥4		1112 (5.3)		23 (7.0)		28 (11.1)		1061 (5.2)	
≥1 Previous fetal loss	21,174	4239 (20.0)	327	59 (18.0)	255	34 (13.3)	20,591	4146 (20.1)	< 0.001
≥1 Previous infant death	21,174	3149 (14.9)	328	55 (16.8)	255	53 (20.8)	20,591	3041 (14.8)	< 0.001
Place of delivery	21,160		327		255		20,578		0.012
Home		19,191 (90.7)		297 (90.8)		245 (96.1)		18,649 (90.6)	
Facility		1933 (9.1)		28 (8.6)		10 (3.9)		1895 (9.2)	
Elsewhere		36 (0.2)		2 (0.6)		0 (0.0)		34 (0.2)	
Gestational age, wk	20,262	38.77 ± 2.93	307	36.06 ± 4.39	242	37.56 ± 3.55	19,713	38.82 ± 2.87	< 0.001
Household characteristics									
Electricity	21,151	4143 (19.6)	328	51 (15.6)	253	40 (15.8)	20,571	4052 (19.7)	0.06
Living standards index^5^	21,141	–0.07 ± 0.94	327	–0.34 ± 0.81	253	–0.37 ± 0.84	20,561	–0.07 ± 0.94	< 0.001

1 Missing data out of possible *n* = 328, 255, and 20,591 in neonatal deaths, postneonatal deaths, and alive infants, respectively, are as follows: age (*n *= 0, *n *= 0, *n *= 11), literacy (*n *= 11, *n *= 2, *n *= 21), education (*n *= 1, *n *= 2, *n *= 25), parity (*n *= 1, *n *= 2, *n *= 18), previous fetal loss (*n *= 1, *n *= 0, *n *= 0), previous infant death (*n *= 0, *n *= 0, *n *= 0), electricity (*n *= 0, *n *= 2, *n *= 20), and living standards index (*n *= 1, *n *= 2, *n *= 30)

2 Deaths were classified as neonatal if they occurred ≤28 d after birth.

3 Deaths were classified as postneonatal if they occurred 29–365 d after birth.

4 *P* values based on χ^2^ test for categorical variables and 1-factor ANOVA for continuous distributions across mutually exclusive groups (neonatal, postneonatal, and alive).

5 Based on derived index for rural northwestern Bangladesh (40).

Birth length, weight, MUAC, HC, CC, and Ponderal Index were lowest in newborns who died as neonates, next lowest in newborns who survived their first month but died postneonatally, and highest in infants who survived their first year. Rates of preterm birth and low birth weight were 54%, 33%, and 19% and 75%, 63%, and 42% among the 3 groups, respectively. Approximately 63% of infants in each group were small for gestational age (**Table 2**). Proportionality across birth size measurements was evident in correlations that ranged from 0.34 (for HC-WLZ) to 0.88 (for CC-weight), a notable exception being a lack of discernable correlation between WLZ and either length or LAZ (**Supplemental Table 2**).

**TABLE 2:** Newborn and maternal size of singletons measured ≤72 h after birth, by infant vital status at 365 d^1^

Characteristic	Total (*n* = 21,174)		Died				Alive (*n* = 20 591)		*P* value^4^
			Neonatal^2^ (*n* = 328)		Postneonatal^3^ (*n* = 255)				
	*n*	*n* (%) or mean ± SD	*n*	*n* (%) or mean ± SD	*n*	*n* (%) or mean ± SD	*n*	*n* (%) or mean ± SD	
Male	21,174	10,798 (51.0)	328	176 (53.7)	255	122 (47.8)	20, 591	10,500 (51.0)	0.378
Age at birth anthropometry, h	21,174	15.0 ± 12.6	328	14.4 ± 12.0	255	15.1 ± 12.2	20, 591	15.1 ± 12.6	0.626
Birth size^5^									
Length, cm	20,773	46.57 ± 2.22	287	43.25 ± 3.75	243	45.28 ± 2.80	20, 243	46.63 ± 2.14	< 0.001
Weight, g	21,172	2558 ± 410	328	1996 ± 611	255	2316 ± 465	20, 589	2570 ± 399	< 0.001
MUAC, cm	21,127	9.51 ± 0.84	309	8.55 ± 1.13	254	9.14 ± 0.97	20, 564	9.53 ± 0.82	< 0.001
Head circumference, cm	21,007	32.59 ± 1.54	307	30.42 ± 2.75	249	31.83 ± 1.95	20, 451	32.63 ± 1.48	< 0.001
Chest circumference, cm	21,042	30.82 ± 2.04	305	27.76 ± 3.21	249	29.76 ± 2.36	20, 488	30.88 ± 1.98	< 0.001
Ponderal index^6^	20,773	25.22 ± 2.42	287	24.17 ± 2.94	243	24.76 ± 2.38	20, 243	25.24 ± 2.40	< 0.001
Preterm (GA <37 wk)	20,262	4011 (19.8)	307	166 (54.1)	242	80 (33.1)	19, 713	3765 (19.1)	< 0.001
Low birth weight (<2500 g)	21,172	9084 (42.9)	328	247 (75.3)	255	161 (63.1)	20, 589	8676 (42.1)	< 0.001
Small for gestational age^7^	20,260	12 884 (63.6)	307	192 (62.5)	242	151 (62.4)	19, 711	12 541 (63.6)	0.859
Maternal size^8^									
Weight, kg	21,092	43.07 ± 6.01	325	41.71 ± 5.47	253	41.83 ± 5.79	20, 514	43.11 ± 6.01	< 0.001
Height, cm	21,083	149.58 ± 5.19	326	148.60 ± 5.49	252	148.49 ± 5.61	20, 505	149.61 ± 5.17	< 0.001
MUAC, cm	21,100	23.39 ± 2.15	326	22.97 ± 1.93	253	23.02 ± 2.07	20, 521	23.40 ± 2.16	< 0.001

1 GA, gestational age; MUAC, midupper-arm circumference.

2 Deaths were classified as neonatal if they occurred ≤28 d after birth.

3 Deaths were classified as postneonatal if they occurred 29–365 days after birth.

4 *P* values based on χ^2^ test for categorical variables and 1-factor ANOVA for continuous distributions across mutually exclusive groups (neonatal, postneonatal, and alive).

5 Missing values for not measurement or out of biologically acceptable range: *n *= 401 in length, *n *= 2 in weight, *n *= 47 in MUAC, *n *= 167 in head circumference, and *n *= 132 in chest circumference.

6 Ponderal index is an indicator of wasting or adequacy of weight adjusted for length calculated as weight in kilograms divided by height in meters cubed (50).

7 Post hoc analysis with small for gestational age defined as a birth weight <10th percentile of an Intergrowth-21st standard growth reference (51).

8 Maternal anthropometry collected at time of enrollment late in the first or early in the second trimester of pregnancy.

Mothers of infants who died throughout the first year were ∼1.35 kg lower in weight and ∼1 cm shorter in height than mothers of surviving infants (Table 2).

**FIGURE 2:**
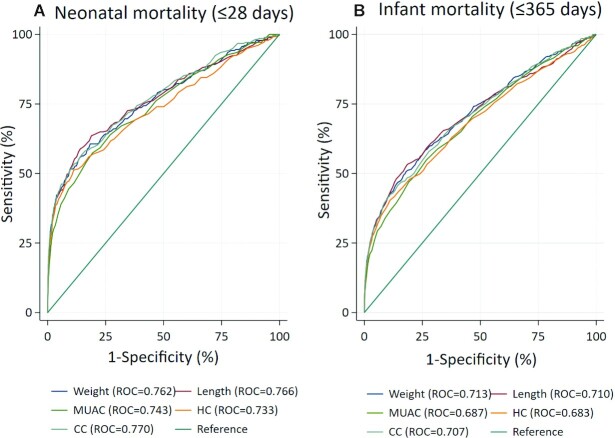
Receiver operating characteristic (ROC) for birth anthropometric measures as predictors of (A) neonatal and (B) infant mortality in singletons measured ≤72 h after birth (*n* = 21,174). CC, chest circumference; HC, head circumference; MUAC, midupper arm circumference.

The ROC analysis revealed comparable discriminatory power to predict mortality across all anthropometric measurements for neonatal (AUC = 0.733–0.770) and infant (AUC = 0.683–0.713) mortality (**Figure 2**). The optimal cutoffs for the five anthropometric measures, based on the Youden Index (sensitivity + specificity – 1) for neonatal mortality, were 44.4 cm for length, 2220 g for weight, 8.8 cm for MUAC, 30.9 cm for HC, and 28.5 cm for CC (**Table 3**). For infant mortality through 365 days, corresponding cutoffs were similar to those for neonatal mortality: 44.9 cm for length, 2220 g for weight, 9.0 cm for MUAC, 30.9 cm for HC, and 28.5 cm for CC, representing differences of 0.5 cm, 0 g, 0.2 cm, 0 cm, and 0 cm, respectively. Thus, the cutoffs were harmonized to 44.5 cm for length, 2200 g for weight, 9.0 cm for MUAC, 31.0 cm for HC, and 28.5 cm for CC to facilitate adoption of 1 cutoff to predict either neonatal or infant mortality. Specificity and PPV for neonatal mortality were highest for CC (88.5% and 11.3%) and HC (86.8% and 8.7%), followed by length (84.6% and 7.1%), weight (82.7% and 6.8%), and lowest for MUAC (73.0% and 3.7%). The same order in measurement specificity and PPV was observed for infant mortality, with values ranging from 88.7% to 73.2% and 16.3% to 6.4%, respectively (Table 3).

**TABLE 3:** Sensitivity, specificity, positive and negative predictive values, and AUC for predicting neonatal and infant mortality using optimal cutoffs for anthropometric measures in singletons measured ≤72 h after birth (*n* = 21,174)^1^

Characteristic		Cutoff	Total	No. died	Alive	Rate^2^	Sensitivity	Specificity	Youden Index	PPV^3^	NPV^4^
Neonatal mortality (≤8 d)											
Length, cm	Optimal	(44.4)	20,773	287	20,486	20.1	60.0	85.9	45.9	8.0	99.1
	Harmonized	44.5	20,773	287	20,486	19.2	60.6	84.6	45.2	7.1	99.1
Weight, g	Optimal	(2220)	21,172	328	20,844	19.6	61.3	81.4	42.7	6.2	99.1
	Harmonized	2200	21,172	328	20,844	20.8	59.5	82.7	42.2	6.8	99.0
MUAC, cm	Optimal	(8.8)	21,127	309	20,818	20.3	58.3	80.6	38.9	5.9	98.9
	Harmonized	9.0	21,127	309	20,818	15.8	65.0	73.0	38.0	3.7	99.2
HC, cm	Optimal	(30.9)	21,007	307	20,700	24.5	53.4	88.7	42.1	10.6	98.7
	Harmonized	31.0	21,007	307	20,700	22.9	53.7	86.8	40.5	8.7	98.8
CC, cm	Optimal	(28.5)	21,042	305	20,737	25.6	55.7	88.5	44.2	11.3	98.7
	Harmonized	28.5	21,042	305	20,737	25.6	55.7	88.5	44.2	11.3	98.7
Infant mortality (≤365 d)											
Length, cm	Optimal	(44.9)	20,773	530	20,243	32.6	54.0	81.3	35.3	8.9	98.1
	Harmonized	44.5	20,773	530	20,243	37.9	50.0	84.8	34.8	11.5	97.7
Weight, g	Optimal	(2220)	21,172	583	20,589	38.2	52.3	81.8	34.1	10.2	97.8
	Harmonized	2200	21,172	583	20,589	40.0	50.9	83.0	33.9	11.1	97.6
MUAC, cm	Optimal	(9.0)	21,127	563	20,564	31.8	56.0	73.2	29.2	6.4	98.1
	Harmonized	9.0	21,127	563	20,564	31.8	56.0	73.2	29.2	6.4	98.1
HC, cm	Optimal	(30.9)	21,007	556	20,451	44.9	42.4	88.9	31.3	15.3	97.0
	Harmonized	31.0	21,007	556	20,451	42.7	43.7	87.0	30.7	13.0	97.2
CC, cm	Optimal	(28.5)	21,042	554	20,488	47.8	43.9	88.7	32.6	16.3	96.9
	Harmonized	28.5	21,042	554	20,488	47.8	43.9	88.7	32.6	16.3	96.9

1 Optimal cutoff points based off Youden Index (35). CC, chest circumference; HC, head circumference; MUAC, midupper-arm circumference; NPV, negative predictive value; PPV, positive predictive value.

2 Deaths per 1000 live births.

3 ${\rm{PPV}} = \frac{{{\rm{sensitivity}} \times {\rm{predicted}}\,{\rm{mortality}}}}{{{\rm{sensitivity}} \times {\rm{predicted}}\,{\rm{mortality}} + (1 - {\rm{specificity}}) \times (1 - {\rm{predicted}}\,{\rm{mortality}})}}$

4 ${\rm{NPV}} = \frac{{{\rm{specificity}} \times (1 - {\rm{predicted}}\,{\rm{mortality}})}}{{(1 - {\rm{sensitivity}}) \times {\rm{predicted}}\,{\rm{mortality}} + {\rm{specificity}} \times (1 - {\rm{predicted}}\,{\rm{mortality}})}}$

Among infants with birth measurements below the 5 harmonized cutoffs, the highest neonatal mortality risk was associated with a newborn CC <28.5 cm (RRR = 9.68; 95% CI: 7.84, 11.94), followed by HC <31.0 cm (8.49; 6.86,10.49), length < 44.5cm (8.66; 6.83, 10.97), weight <2200 g (6.57; 5.31, 8.13), and MUAC <9.0 cm (5.01; 4.03,6.23) (**Table 4**). Rates of mortality to 365 days were higher and RRR lower (range: 3.40–6.02) for newborns with birth measurements below the same respective cutoffs; however, RRRs were in the same order as observed for neonatal mortality. Notably, neonatal mortality rate and infant mortality rate were within very narrow ranges among newborns whose measurements were greater than or equal to harmonized cutoffs, at 6.5–7.9 and 15.3–17.3 per 1000 live births, respectively.

**TABLE 4.** Relative risk ratio of neonatal and infant mortality (≤365 d) for harmonized anthropometric cutoffs^1^

Measurement cutoff	Neonatal mortality (≤28 d)		Infant mortality (≤365 d)	
	Mortality rate^2^ (number of deaths/number of measures)	Relative risk ratio^3^ (95% CI)	Mortality rate^2^ (number of deaths/number of measures)	Relative risk ratio^3^ (95% CI)
Length (reference: ≥44.5 cm)	6.5 (115/17,713)	1.00	15.3 (271/17,713)	1.00
<44.5 cm	56.2 (172/3060)	8.66 (6.83, 10.97)	84.6 (259/3060)	5.53 (4.69, 6.53)
Weight (reference: ≥2200 g)	7.9 (138/17,504)	1.00	16.7 (293/17504)	1.00
<2200 g	51.8 (190/3668)	6.57 (5.31, 8.13)	79.1 (290/3668)	4.72 (4.06, 5.50)
MUAC (reference: ≥9.0 cm)	7.6 (123/16,229)	1.00	17.1 (278/16,229)	1.00
<9.0 cm	38.0 (186/4898)	5.01 (4.03, 6.23)	58.2 (285/4898)	3.40 (2.89, 3.99)
HC (reference: ≥31.0 cm)	7.7 (143/18,506)	1.00	17.3 (320/18,506)	1.00
<31.0 cm	65.6 (164/2501)	8.49 (6.86, 10.49)	94.4 (236/2501)	5.46 (4.63, 6.43)
CC (reference: ≥28.5 cm)	7.4 (139/18,731)	1.00	17.0 (318/18,731)	1.00
<28.5 cm	71.8 (166/2311)	9.68 (7.84, 11.94)	102.1 (236/2311)	6.02 (5.15, 7.02)

1 Missing values for not measurement or out of biologically acceptable range: *n *= 401 in length, *n *= 2 in weight, *n *= 47 in MUAC, *n *= 167 in HC, and *n *= 132 in CC. CC, chest circumference; HC, head circumference; MUAC, midupper-arm circumference.

2 Number of deaths per 1000 live births.

3 Relative risk ratio was produced by general linear models.

**FIGURE 3:**
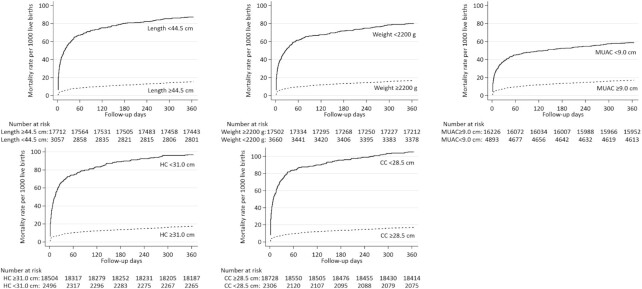
Nelson–Aalen cumulative hazard curves for infants through age 12 mo (365 d) by harmonized cutoffs at birth for length, weight, and midupper arm circumference (MUAC), head circumference (HC), and chest circumference (CC).

**Figure 3** presents Nelson–Aalen cumulative hazard curves depicting the mortality experience of infants by the number of days since assessment for each measurement, revealing the sharpest rise in mortality within 60 d. The highest sustained mortality rate thereafter is in infants whose CC was <28.5 cm compared with higher, relative to other measurements at their harmonized cutoffs. Figure 3 also reveals the far more uniformly favorable and comparable survival of infants whose values were above the harmonized cutoffs for all birth measurements.

Given the opportunity to measure multiple anthropometric measurements, we also explored if any 2 measures at harmonized cutoffs might further improve the prediction of mortality risk (**Table 5**and**Supplemental Table 3**). Risk and predictability of neonatal mortality was highest among children with both an HC <31.0 cm and a CC <28.5 cm (RRR = 15.74; 95% CI: 12.54, 19.75; PPV = 10.7%), relative to children whose HC and CC were both at or above these respective cutoffs. A combined length <44.5 cm and CC <28.5 cm identified newborns at the next highest risk of neonatal mortality, followed by a length <44.5 cm and HC <31.0 cm. Except for a combined MUAC <9.0 cm and weight <2200 g, all other paired measurements below their harmonized cutoffs were associated with RRR ≥10 compared with having both measures above their respective cutoffs. Risk of infant mortality to 365 d associated with paired measurements largely mirrored those observed with neonatal mortality, although with higher PPVs. The highest risk was evident among newborns with an HC <31.0 cm plus CC <28.5 cm (RRR = 9.19; 95% CI: 7.72, 10.95; PPV = 14.5%), followed by a combination of length <44.5 cm plus CC <28.5 cm and length <44.5 cm plus HC <31.0 cm.

**TABLE 5:** Relative risk ratio (95% CI) and positive predictive values of neonatal (≤28 d) and infant mortality (≤365 d) using harmonized cutoffs for any 2 anthropometric measurements combined^1^

Characteristic	Length ≥44.5 cm	Length <44.5 cm	Weight ≥2200 g	Weight <2200 g	MUAC ≥9.0 cm	MUAC <9.0 cm	HC ≥31.0 cm	HC <31.0 cm
Neonatal mortality (≤28 d)								
Length ≥44.5 cm	—	—						
Length < 44.5 cm	—	—						
Weight ≥2200 g	1.00 (reference)	3.19 (1.93, 5.28), 2.0	—	—				
Weight <2200 g	1.29 (0.70, 2.41), 0.8	10.99 (8.60, 14.06), 7.0	—	—				
MUAC ≥9.0 cm	1.00 (reference)	3.30 (2.04, 5.33), 2.1	1.00 (reference)	2.58 (1.44, 4.59), 1.8	—	—		
MUAC <9.0 cm	1.28 (0.77, 2.14), 0.8	11.43 (8.93, 14.63), 7.1	1.68 (1.04, 2.73), 1.2	8.00 (6.37, 10.05), 5.6	—	—		
HC ≥31.0 cm	1.00 (reference)	3.30 (2.15, 5.05), 2.1	1.00 (reference)	1.93 (1.26, 2.97), 1.4	1.00 (reference)	1.83 (1.24, 2.70), 1.24	—	—
HC <31.0 cm	1.79 (0.93, 3.44), 1.1.	14.34 (11.24, 18.28), 9.0	1.61 (0.78, 3.31), 1.14	12.26 (9.75, 15.43), 8.7	2.84 (1.68, 4.82), 1.93	12.24 (9.68, 15.47), 8.3	—	—
CC ≥28.5 cm	1.00 (reference)	3.20 (2.13, 4.81), 2.0	1.00 (reference)	1.75 (0.57, 5.42), 1.2	1.00 (reference)	1.33 (0.85, 2.10), 1.6	1.00 (reference)	2.29 (1.39, 3.76), 1.56
CC <28.5 cm	2.18 (1.11, 4.28), 1.3	14.57 (11.41, 18.60), 9.0	1.54 (0.92, 2.56), 1.1	11.09 (8.89, 13.83), 7.9	2.31 (0.97, 5.51), 0.9	10.99 (8.79, 13.75), 7.8	3.35 (2.16, 5.20), 2.3	15.74 (12.54,19.75), 10.7
Infant mortality (≤365 d)								
Length ≥44.5 cm	—	—						
Length <44.5 cm	—	—						
Weight ≥2200 g	1.00 (reference)	2.60 (1.79, 3.79), 3.8	—	—				
Weight <2200 g	1.58 (1.09, 2.30), 2.3	6.98 (5.88, 8.27), 10.2	—	—				
MUAC ≥9.0 cm	1.00 (reference)	2.92 (2.12, 4.02), 4.3	1.00 (reference)	2.61 (1.83, 3.71), 4.2	—	—		
MUAC <9.0 cm	1.25 (0.92, 1.71), 1.9	6.98 (5.84, 8.35), 10.3	1.27 (0.90, 1.81), 2.0	5.34 (4.51, 6.31), 8.5	—	—		
HC ≥31.0 cm	1.00 (reference)	2.84 (2.15, 3.76), 4.2	1.00 (reference)	2.15 (1.67, 2.77), 3.3	1.00 (reference)	1.68 (1.30, 2.17), 2.6	—	—
HC <31.0 cm	1.75 (1.15, 2.65), 2.6	8.47 (7.08, 10.13), 12.5	1.92 (1.22, 3.03), 3.0	7.70 (6.46, 9.17), 12.0	2.82 (1.99, 3.99), 4.4	7.34 (6.13, 8.81), 11.4	—	—
CC ≥28.5 cm	1.00 (reference)	2.89 (2.17, 3.84), 2.0	1.00 (reference)	2.11 (1.06, 4.19), 3.3	1.00 (reference)	1.34 (1.00, 1.80), 4.3	1.00 (reference)	2.15 (1.54, 3.00), 3.4
CC <28.5 cm	2.49 (1.67, 3.70), 1.3	8.62 (7.23, 10.27), 9.0	1.96 (1.45, 2.65), 3.1	7.01 (5.94, 8.25), 11.0	2.63 (1.56, 4.45), 2.2	6.73 (5.70, 7.96), 10.9	2.77 (2.05, 3.72), 4.4	9.19 (7.72, 10.95), 14.5

1 Missing values for not measurement or out of biologically acceptable range: *n *= 401 in length, *n *= 2 in weight, *n *= 47 in MUAC, *n *= 167 in HC, and *n *= 132 in CC. Values are relative risk ratio (95% CI), positive predictive value (%). Relative risk ratio was produced by general linear models. CC, chest circumference; HC, head circumference; MUAC, midupper-arm circumference.

## Discussion

This study assessed the discriminatory power of anthropometry taken within 72 h of birth to predict all-cause neonatal and infant mortality in a population cohort of over 21,000 singletons in rural Bangladesh. We identified best cutoffs, harmonized to predict both neonatal and infant mortality, to be 44.5 cm for length, 2200 g for weight, and 9.0 cm, 31.0 cm, and 28.5 cm for midupper arm, head, and chest circumferences, respectively.

Specificity, a metric that reflects the percentage of correctly classified infants who survived to 28 and 365 d, ranged from 83% to 88% for all birth measurements except for MUAC, for which specificity was 73% for both outcomes. RRRs for both mortality rates below their harmonized cutoffs were highest for chest circumference, followed by length and head circumference, which were comparable, weight, and lastly MUAC. Positive predictive value, which reflects the percentage of infants with measurements below a harmonized cutoff who died, was highest for chest circumference, predicting 11% and 16% of all neonatal and total infant deaths, followed by head circumference, length, weight, and MUAC. These findings emphasize the potential of all assessed birth measurements to identify infants at risk of dying during the first year of life; however, a novel finding is the superior predictive power of a chest circumference, a rarely measured dimension obtainable with an insertion tape (8). Superiority of this measurement in predicting infant mortality might be reflecting anatomic specificity to the size of the chest cavity and consequent lung size and health (41), noting the importance of acute respiratory infections as major causes of death in infancy (42).

A second novel aspect of this study was to examine the capability of 2 newborn measurements to predict infant mortality. Every index comprising both measurements lying below their respective, harmonized cutoffs was associated with a higher RRR (range: 5.34–9.19) than either single measurement in a pair (range: 3.40–6.02). The strongest predictive value emerged from an index that combined newborn chest and head circumferences at cutoffs of <31.0 cm and <28.5 cm that, when compared with infants with measured values above both cutoffs, yielded the highest RRR for neonatal mortality of 15.74. The same pair of circumferential measurements remained superior in predicting infant mortality through 12 mo of age (RRR = 9.19). Importantly, both of these measurements are taken with a single, extended insertion tape (8). Newborns with a discordant classification, whereby only 1 of 2 paired measurements were below a harmonized cutoff, were either slightly higher or comparable in risk of mortality than infants for whom both paired measurements were above their respective cutoffs. These findings are consistent with reports elsewhere showing symmetric compared with asymmetric fetal growth restriction posing a higher risk of neonatal mortality (43).

Globally, a weight <2500 g is considered the standard cutoff for classifying a low birth weight (44). Yet, based on AUC, RRR, and PPV analyses, among newborns in this large rural cohort in Bangladesh, a birth weight <2200 g (AUC = 0.67, RRR = 4.72, PPV = 11.1%) appeared superior in predicting newborn mortality than a birth weight <2500 g (AUC = 0.64, RRR = 3.10, PPV = 3.30) (data not shown), suggesting that local adjustment of a birth weight cutoff may be preferred to the conventional cutoff when screening newborns for mortality risk.

MUAC was surprisingly less predictive of mortality than either chest or head circumference, given a low MUAC (<9.0 cm) at birth has been shown to be comparable to low birth weight in predicting neonatal mortality in India (16) and Guatemala (17). Furthermore, in Nepal, MUAC measured throughout early infancy (45) and the preschool years (46) has been strongly associated with the risk of mortality. However, none of these studies compared the predictiveness of MUAC with other concurrently assessed circumferential measurements.

The data collected in this study provided an opportunity to concurrently assess the performance of multiple dimensions of birth size, obtained by highly standardized methods, in predicting infant mortality in a large, population-based birth cohort setting typical of rural Bangladesh (32). Cutoffs and associated levels of risk for neonatal and infant mortality were based on actual, unadjusted measurement distributions, facilitating their direct adoption by primary care workers to use the harmonized cutoffs for screening high-risk infants.

Our study was large, population based, highly standardized, and conducted in an area that typifies rural Bangladesh (33). However, there are limitations to note. Most important, while reaching newborns for anthropometry within 72 h is a common research practice in remote settings where most births occur at home (47), our study reveals the vital consequence of any delay in reaching newborns. Although staff reached infants ∼16 h after birth, including one-fourth within 8 h, 653 infants died prior to the home visit, comprising 63% of all neonatal deaths. Consequently, as very early neonatal deaths are most likely to occur among preterm and growth-restricted infants (48, 49), mortality risks associated with newborn size derived from this analysis are likely to be underestimates, although relevant for health care services reaching newborns the day after birth or later. On the other hand, our findings that mothers of infants who died shortly after birth, and thus not measured, were younger and shorter, were more likely to be preterm, and had obstructed labor and delivered in a health facility (Supplemental Table 1) suggests that missed birth anthropometry in the home can be partially compensated by incorporating standardized birth assessment in health facilities.

In conclusion, although weight is the most frequent and, usually, the only measurement taken at birth, we demonstrate in a rural Bangladesh setting that measurements of length and head, chest, and arm circumference can also be deployed with harmonized, optimized cutoffs to assess risk of mortality throughout infancy. Furthermore, combining information from any 2 measurements can further enhance mortality risk prediction. Among measurements tested in this study, chest circumference, alone and in combination with head circumference, best predicted neonatal and infant mortality.

## Supplementary Material

nqab432_Supplemental_File

## Data Availability

Data described in the manuscript, code book, and analytic code will be made available upon request pending approval by the authors.

## Notes

Supported by the Bill & Melinda Gates Foundation (grants GH614 and OPP1441435) and the Sight and Life Global Nutrition Research Institute.

PC is an Associate Editor of *AJCN* and played no role in the journal's evaluation of the manuscript.

Supplemental Tables 1–3 are available from the “Supplementary data” link in the online posting of the article and from the same link in the online table of contents at https://academic.oup.com/ajcn/.

Abbreviations used: CC, chest circumference; HC, head circumference; LAZ, length-for-age *z* score; MUAC, midupper arm circumference; PPV, positive predictive values; ROC, receiver operating characteristic; RRR, relative risk ratio; WLZ, weight-for-length *z* score.
